# CAN *Stryphnodendron adstringens* EXTRACT IMPROVE THE RESULTS OF FISTULOTOMY FOLLOWED BY PRIMARY SPHINCTEROPLASTY IN THE TREATMENT OF TRANSSPHINCTERIC FISTULAE?

**DOI:** 10.1590/0102-672020200003e1540

**Published:** 2020-12-18

**Authors:** Adriana de Souza RÓS, Carlos Henrique Marques dos SANTOS, Doroty Mesquita DOURADO, Moisés Soares da SILVA-NETO, Isabela CALDEIRA, Leandro de Oliveira FURTADO

**Affiliations:** 1 Colon and Rectum Department, Universitary Hospital Maria Aparecida Pedrossian, Federal University of Mato Grosso do Sul, Campo Grande, MS, Brazil; 2 Anhanguera-Uniderp University, Campo Grande, MS, Brazil

**Keywords:** Rectal fistula, Stryphnodendron adstringens, Fecal incontinence, Colorectal surgery, Fístula retal, Stryphnodendron adstringens, Incontinência fecal, Cirurgia colorretal

## Abstract

**Background:**

There is still a need for progress in the treatment of transsphincteric anal fistulae and the use of herbal medicines seems promising.

**Aim:**

To evaluate the efficacy of *Stryphnodendron adstringens* associated with fistulotomy and primary sphincteroplasty in the treatment of transsphincteric fistulae in rats.

**Methods:**

Thirty Wistar rats were used, which were submitted to transsphincteric fistulas with steel wire 0; after 30 days a treatment was performed according to the group. Group A (n=10) was submitted to fistulotomy; group B (n=10), fistulotomy followed by primary sphincteroplasty with “U” stitch with polyglactin 911 4-0; group C(n=10) , similar to group B, but with the interposition between the muscular stumps of hemostatic sponge soaked in *Stryphnodendron adstringens* extract. Euthanasia was performed after 14 days, resecting a segment of the anal canal for histological analysis, which aimed to evaluate the closure of the fistula, the area of separation of the muscle cables, the inflammatory process and the degree of fibrosis.

**Results:**

No animal had a remaining fistulous tract. About the spacing between the muscle cables, an average of 106.3 µm^2^ was observed in group A, 82.8 µm^2^ in group B and 51.8 µm^2^ in group C (p<0.05). There was no difference between the groups regarding the inflammatory process and, in relation to fibrosis, in group A there was a mean of 0.6, in group B 0.7 and in group C 0.2 (p<0.05).

**Conclusions:**

*Stryphnodendron adstringens* extract was able to allow less spacing between muscle cables in rats submitted to fistulotomy followed by primary sphincteroplasty, in addition to providing less local fibrosis.

## RESUMO

**Racional:**

Há ainda necessidade de avanço no tratamento das fístulas transesfincterianas e o uso de fitoterápicos parece promissor.

**Objetivo:**

Avaliar a eficácia da *Stryphnodendron adstringens* associada à fistulotomia e esfincteroplastia primária no tratamento de fístulas transesfincterianas em ratos.

**Métodos:**

Utilizou-se 30 ratos Wistar submetidos à confecção de fístulas transesfincterianas com fio de aço 0; após 30 dias realizou-se tratamento de acordo com o grupo. O grupo A (n=10) foi submetido à fistulotomia; o grupo B (n=10) fistulotomia seguida de esfincteroplastia primária com ponto em “U” com poliglactina 911 4-0; o grupo C (n=10), semelhante ao grupo B, porém com a interposição entre os cotos musculares de esponja hemostática embebida em extrato de *Stryphnodendron adstringens*. Realizou-se eutanásia após 14 dias, ressecando-se segmento do canal anal para análise histológica, que objetivou avaliar o fechamento da fístula, a área de afastamento dos cabos musculares, o processo inflamatório e o grau de fibrose.

**Resultados:**

Nenhum animal apresentou trajeto fistuloso remanescente. Quanto ao afastamento entre os cabos musculares observou-se média 106,3 µm^2^ no grupo A, 82,8 µm^2^ no grupo B e 51,8 µm^2^ no grupo C (p<0,05). Não houve diferença entre os grupos quanto ao processo inflamatório e, em relação à fibrose; no grupo A observou-se média 0,6, no grupo B 0,7 e no grupo C 0,2 (p<0,05).

**Conclusões:**

O extrato de *Stryphnodendron adstringens* foi capaz de permitir menor afastamento entre os cabos musculares em ratos submetidos à fistulotomia seguida por esfincteroplastia primária, além de proporcionar menor fibrose local.

## INTRODUCTION

The anal fistula is the second most common anorectal disorder, behind hemorrhoidal disease^6^ ^,^ ^25^, with intersphincteric type being the most common; however, transsphincteric fistulae are more challenging in terms of therapeutic approach, as they are associated with a greater number of patients who develop fecal incontinence after surgical treatment^16^ ^,^ ^19^. 

The medical literature demonstrates that transsphincteric fistulae present a risk of recurrence ranging from 0.7% to 26.5%^12^ ^,^ ^27^, and 30% to 35% of some reduction in sphincter function^1^ ^,^ ^4^ ^,^ ^26^. 

Among the various techniques used in the treatment of transsphincteric fistula, the advancement of the mucosal flap and the fistulotomy followed by primary sphincteroplasty stand out. Although there are techniques with better results in terms of continence, such as LIFT (ligation of intersphincteric fistula tract), plugs and biological glue, it should be noted that these have a high cost and were used in a much smaller number than the first ones^1^ ^,^ ^12^ ^,^ ^27^.

Fistulotomy has historically shown the best result in the treatment of anal fistula, with lower rates of recurrence. In the case of transsphincteric fistulae, however, the result can be catastrophic with regard to continence, which can be solved by immediate sphincteroplasty. As long as there is no dehiscence, there may be adequate sphincter function, and there is no technical alternative that can minimize this risk, except for proper cleaning of the wound, tension-free suture and use of appropriate material^18^.

The Kshara Sutra is an alternative outpatient treatment method with the use of drugs in order to cure the anal fistula, providing preserved anal continence and a 3.33% disease recurrence rate, with even less pain, fewer complications and low cost^4^ ^,^ ^21^. However, this therapy is rarely used in the West.

Barbatimão (*Stryphnodendron adstringens*), a species of the Fabaceae family, genus Mimosoide, is native to the Brazilian cerrado region^17^. The use of barbatimão in folk medicine is common, and the bark, leaves and roots can be used for the formulation of extracts. The high tannin content present in the extracts provides an astringent, antiseptic, antioxidant and healing action^2^. The main medicinal effect attributed to this plant is the healing power^9^. 

Considering that there is still a need for advancement in the treatment of transsphincteric anal fistulae, since the current therapeutic possibilities are still below what is desired in terms of recurrence and incontinence, the use of herbal medicines with the possibility of anti-inflammatory action has grown, as demonstrated in relation to *Stryphnodendron adstringens*, it is essential to investigate the association of surgical treatment with this product to verify the therapeutic response. 

The purpouse of this research was to evaluate the efficacy of *Stryphnodendron adstringens* associated with fistulotomy and primary sphincteroplasty in the treatment of transsphincteric fistula in rats.

## METHODS

The study was approved by the Animal Use Ethics Committee of the Federal University of Mato Grosso do Sul and all the rules established by the Brazilian College of Animal Experimentation were followed.

Thirty adult male Wistar rats, weighing approximately 300 g each, were used, which were kept in suitable cages with food and water ad libitum, 12-hour light and dark cycles, at a controlled temperature of 23º C in the vivarium of the Federal University of Mato Grosso do Sul, Campo Grande, MS, Brazil.

The animals were anesthetized peritoneally to make anal fistulas using xylazine hydrochloride 2%, at a dose of 10 mg/kg, and ketamine hydrochloride 10%, at a dose of 50 mg/kg, in a proportion of 2:1, using 0.1 ml of the solution for every 100 g of weight.

**FIGURE 1:**
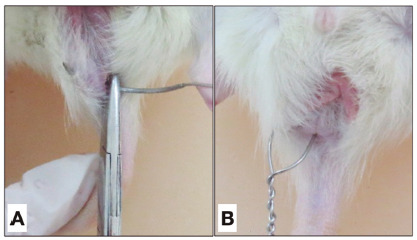
A) Transection of the anal sphincter with steel wire; B) steel wire positioned for creating the fistula tract.

After anesthetization, anal fistulae were made with steel wire number 0 (Aciflex^®^), transfixing the anal sphincter, with the needle being introduced into the dentated line in the right lateral position and externalized approximately 1 cm laterally to the right anal margin (Figure 1A). The steel wire was cut and twisted (Figure 1B), being left for 30 days.

The animals were then kept in individual cages with the care described above for 30 days, until the second part of the experiment was performed, when they were again anesthetized and subjected to treatment according to the groups constituted: group A (n=10): fistulotomy; group B (n=10): fistulotomy + sphincteroplasty; group C (n=10): fistulotomy + sphincteroplasty + *Stryphnodendron adstringens*.

After anesthesia, all animals were submitted to a fistulotomy with a cold-scalpel n^o^. 15 on the steel wire, which was then removed and curettage was then performed. All animals underwent hemostasis by compressing the wound with sterile gauze for 10 min. In group A, this was the only procedure performed. In group B, after hemostasis, primary sphincteroplasty with a “U” stitch was performed, joining the muscle cables using polyglactin 911 4-0 thread, with the skin kept open. In group C, the same procedure described for group B was performed, however prior to the fixation of the suture, a previously cut sterile hemostatic sponge (Gelfoam^®^) was inserted between the muscle stumps measuring 5x5 mm soaked in *Stryphnodendron adstringens* extract, tying then the thread and also leaving the skin open.

The animals were maintained with the care already mentioned for 14 days, when euthanasia was performed using isoflurane in the lethal dose. Following euthanasia, a segment of the anal canal and perianal skin was resected from where the treatment was performed, in the form of a cube, with a cold-scalpel n^o^ 15, in order to fully include the region operated. The samples were washed with saline solution, placed in separate flasks and identified for the group, soaked in a 10% formaldehyde buffered solution for later histological analysis. Subsequently, the material was processed in increasing alcohol filters, diaphanized in xylol and included in historic paraffin and made in cross sections of 5μm thick with the aid of a rotating microtome (Microm. HM320). As the sections were cut by the H&E and Gomori’s trichrome technique for qualitative histopathological analysis. The digital image capture of slides stained with them were performed in the Carl Zeiss photomicroscope coupled to a Samsung microcamera connected to a computer with an image capture card. The reading and interpretation of the findings were made with the professional without knowledge about the group belonging to each animal. 

Histological analysis was performed considering the following aspects: persistence of the fistulous tract, distance between muscle cables (area), inflammatory infiltrate and fibrosis.

### Persistence or closure of the fistulous tract

Was visualized by microscopy the persistence of the fistula; closure was considered only when the entire tract was closed; maintenance, even if the tract was short, was considered persistence.

### Muscle cable spacing area

Was done under optical microscopy, in a coronal section of the anal canal, in which the area was measured in pixels and converted into square micrometers (µm^2^) after marking with the cursor the entire wall of the fistulous tract.

### Inflammatory infiltrate

Scores were applied according to the count of the inflammatory foci. When no inflammatory focus was observed, score 0 (absent) was determined, from one to two foci, score 1 (mild), from three to four foci, score 2 (moderate), more than four foci, score 3 (intense).

### Fibrosis

Was also assessed by optical microscopy using a score according to the count of collagen fibers present per field: 0, absent; 1, mild; 2, moderate; and 3, intense. 

### Statistical analysis

Fisher’s exact test for a 2x3 contingency table was used to compare the muscle cable spacing between the studied groups. Histological analysis regarding the inflammatory process and fibrosis were evaluated by ANOVA test with post-Turkey test, with a significance level of 5%. Data analysis was performed using the Statistical Package for the Social Sciences 24 (SPSS 24).

## RESULTS

Fistulae closed in all animals, demonstrating that both isolated fistulotomy and associated with primary sphincteroplasty (with and without *Stryphnodendron adstringens* extract) were effective in the treatment of transsphincteric fistula.

**FIGURE 2:**
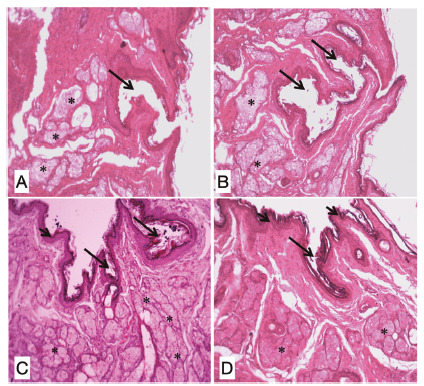
Photomicrograph showing the appearance of the operated region after 14 days: A and B) animals of group A; C) group B; D) group C (H&E x10)

In group A, naturally there was a separation of the muscle cables, since after the fistulotomy no procedure was added, healing the lesion by second intention. In groups B and C, the muscle cables were brought closer after the fistulotomy, but even so, there was some degree of separation of the muscle cables (Figure 2).

*=sebaceous glands; long arrows=spacing between muscle cables; short arrows= structuring the lining tissue

As for the spacing between muscle cables, an average of 106.3 µm^2^ was observed in group A, 82.8 µm^2^ in group B and 51.8 µm^2^ in group C (p<0.05, Table 1).

**TABLE 1:** Assessment of the gap between muscle cables (in µm^2^) between group.

Individual	Group A	Group B	Group C
1	103	88	58
2	101	79	52
3	113	82	64
4	111	89	35
5	102	75	45
6	114	78	53
7	105	87	44
8	101	85	55
9	107	91	51
10	106	74	61
Average±standard deviation	106.3±1.54 A	82.8±6.07 B	51.8±8.65 C

n=10/group; ANOVA test with Tukey’s post-test; different letters indicate significant statistical differences (p<0.05)

The inflammatory process was evaluated in the animals of the three groups without any difference between them. The mean degree of inflammation in group A was 0.4, while in group B it was observed 0.5 and in group C 0.3 (Table 2).

**TABLE 2:** Assessment of the degree of inflammation in the region undergoing treatment according to groups

**Individual**	Group A	Group B	Group C
1	1	0	0
2	0	1	0
3	0	1	0
4	0	0	1
5	1	1	0
6	1	0	0
7	0	1	1
8	1	0	0
9	0	0	0
10	0	1	1
Average	0.4	0.5	0.3

n=10/group; Kruskal-Wallis test; p=0.67

Regarding the intensity of fibrosis determined by the amount of collagen fibers deposited in the healing zone, in group A there was a mean of 0.6, in group B 0.7 and in group C 0.2 (p<0.05, Table 3).

**TABLE 3:** Assessment of the degree of fibrosis in the region undergoing treatment according to the groups

Individual	Group A	Group B	Group C
1	1	1	0
2	0	1	0
3	0	0	0
4	1	1	1
5	1	1	0
6	1	0	0
7	0	1	0
8	0	1	0
9	1	1	0
10	1	0	1
Average	0.6 ^A^	0.7 ^A^	0.2 ^B^

n=10/group; Kruskal-Wallis test; A p>0.05; B p<0.05

## DISCUSSION

Seyfried et al. ^23^ reported that resection of the fistulous tract followed by primary reconstruction of the anal sphincter is the procedure with the best results in transsphincteric fistulae. Of 424 patients with transsphincteric fistulae operated by this technique, they were cured in 88.2%, reaching 95.8% with a second intervention, which is really an excellent result compared to other techniques. However, 23% of patients reported some degree of fecal incontinence in the postoperative period, although the majority was mild. This is the great dilemma of the coloproctologist, offering a technique that achieves a high cure rate but also observing the degree of complications, with incontinence being one of the most feared.

Undoubtedly, the results of fistulotomy followed by primary sphincteroplasty are consistent in terms of the chance of cure, low risk of recurrence, reduced cost, technical ease for specialists and with the great advantage of solving the problem with a single procedure in most cases, allowing thus an earlier return to the usual activities for these patients^5^ ^,^ ^7^ ^,^ ^15^.

However, there is a lack of something that can contribute to the technique to act in its greatest weakness, which is in relation to fecal incontinence. In this regard, with the increasing use of herbal medicines with proven anti-inflammatory and healing action, there could be a beneficial association of methods aimed at minimizing failures in sphincter reconstruction, which could occur as a result of local contamination, infection, dehiscence, and, consequently, damage anatomical and functional to the sphincter.

Barbatimão (*Stryphnodendron adstringens*) has a recognized effect on the healing of skin wounds, biological activity in the treatment of infections, inflammation, antioxidant and antimicrobial activity, probably due to the presence of tannin compounds, especially proanthocyanidins^24^. Freitas et al. ^8^ in a study in rats even observed an antifungal effect exactly due to the polymeric proanthocyanidins present in barbatimão, including species of *Candida albicans* resistant to fluconazole. Theoretically, this action could be useful for the purpose described above, which in fact may have happened.

In the present study, the group treated with *Stryphnodendron adstringens* presented the same average of local inflammation as the other groups; however, it should be considered that the rats have a biological response and healing in different periods than humans, so that after 14 days there was no more moderate or intense inflammation even in the groups that did not use the plant, but perhaps in a shorter period it would be possible to verify difference between the groups. This is because when analyzing the degree of fibrosis, the benefit of *Stryphnodendron adstringens* was evident, since in this group it was lower than in the others. As a higher degree of inflammation may result in more fibrosis^20^, it is inferred that in group C there must have been less inflammation in the previous period. 

In any case, regardless of whether there has been no difference in inflammation, less fibrosis should provide a better functional result for the anal canal, which could benefit the preservation of fecal continence^20^. This could be one of the causes of fecal incontinence after primary sphincteroplasty, since in addition to opening or removing the fistulous tract, curettage, manipulation and suturing of the sphincters, there is expected to be fibrosis resulting from this process. So, although the anatomical aspect at the end of the surgery is satisfactory, in the medium and long term the function may not be adequate. There is no direct proof of this, but there is evidence that the more fibrosis, the greater the functional impairment in the anal canal^13^ ^,^ ^14^ ^,^ ^20^, so that a product that reduces fibrosis, as occurred in the present study with the use of *Stryphnodendron adstringens*, could lead greater preservation of fecal continence.

There is no other publication in the literature that has evaluated *Stryphnodendron adstringens* in the treatment of anal fistulae, but in other types of wounds it has also been shown to reduce inflammation and fibrosis^22^. Henriques et al.^10^ studying *Stryphnodendron adstringens* in an experimental model of arthritis demonstrated that it reduces leukocyte migration and the accumulation of neutrophils at the site of inflammation, thus decreasing the inflammatory process and consequently presenting less fibrosis.

If inflammation and fibrosis are related to continence due to the functional aspect, the removal of muscle cables is directly associated with fecal incontinence due to the anatomical factor, since the sphincter contraction will not be complete if there is a discontinuity in the muscle. Thus, the verification that in the present study there was less separation of muscle cables in group C leads us to imagine that it could provide better sphincter function compared to the group that also underwent primary sphincteroplasty by the same technique, however without association with *Stryphnodendron astringens*. Group A, treated only by fistulotomy, naturally presented the greatest distance from muscle cables compared to the other groups. Fistulotomy, an old technique and probably the most used in the world for the treatment of anal fistulae, still has its role especially because it is associated with low rates of recurrence, but unfortunately it is impractical in most cases of transsphincteric fistula due to the risk of incontinence.

It is known that the inflammatory process and especially the infection can lead to dehiscence, which in the case of a sphincter suture, could be the cause of fecal incontinence after sphincteroplasty^3^. *Stryphnodendron adstringens* has also demonstrated an antimicrobial effect, in addition to its effectiveness in reducing inflammation. Souza-Moreira et al. ^24^ had already demonstrated that the proanthocyanidins, compounds of the tannin, present in the leaves of *Stryphnodendron adstringens* would have antimicrobial action, which could have contributed to fight pathogens present in the anal canal and reduce the consequent removal of muscle cables after sphincter reconstruction, which did not occur in animals not treated by the plant.

We have observed, especially in the last two decades, the emergence of new techniques for the treatment of anal fistulae, such as those already mentioned, LIFT, use of biological glue, plugs and more recently stem cells^11^, however still without consistent results that would allow the replacement of classic techniques such as advancement flap and primary sphincteroplasty for transsphincteric fistulae. Perhaps an alternative way is to improve the good techniques already described, adding something that resolves or minimizes its weaknesses, as we understand to have happened in this experimental research, in which *Stryphnodendron adstringens* acted beneficially in two important stages of the treatment of fistulae: reducing the removal of muscle cables, thus improving the anatomical result, and decreasing local fibrosis, thus potentially improving the functional result. Evidently, there is a lack of analyzes that can confirm the therapeutic potential presented here, such as pressure assessments of the anal canal, which should be the objective of future research, but the results obtained with this paper serve as an initial parameter with promising results of the association of a consecrated operative technique to a phytotherapic widely found in our environment, of low cost and without demonstrated adverse effects.

## CONCLUSIONS

*Stryphnodendron adstringens* extract was able to allow less spacing between muscle cables in rats subjected to fistulotomy followed by primary sphincteroplasty, in addition to providing less local fibrosis.
